# The Effect of the *swnR* Gene on Swainsonine Biosynthesis in *Alternaria oxytropis* OW7.8, an Endophytic Fungus of *Oxytropis glabra*

**DOI:** 10.3390/microorganisms13061326

**Published:** 2025-06-06

**Authors:** Ning Ding, Chang Liu, Ping Lu, Lu Bai, Bo Yuan

**Affiliations:** 1 College of Life Science and Technology, Inner Mongolia Normal University, Hohhot 010022, China; ndingning@163.com (N.D.); ecibxivq@outlook.com (C.L.); bl1434144573@163.com (L.B.); yuanbo934@126.com (B.Y.); 2 Key Laboratory of Biodiversity Conservation and Sustainable Utilization in Mongolian Plateau for College and University of Inner Mongolia Autonomous Region, Hohhot 010022, China; 3 College of Veterinary Medicine, Inner Mongolia Agricultural University, Hohhot 010000, China

**Keywords:** swainsonine, *Alternaria oxytropis* OW7.8, endophytic fungi, gene knockout, *swnR* gene

## Abstract

The *swnR* gene was cloned in the endophytic fungus *Alternaria oxytropis* OW 7.8 isolated from *Oxytropis glabra*, and the gene knockout mutant Δ*swnR* was first constructed in this study. Compared with *A. oxytropis* OW 7.8, the Δ*swnR* exhibited distinct morphological alterations in both colony and mycelial structure, a slower growth rate, and significant reductions in swainsonine (SW) levels, indicating that the function of the *swnR* gene promoted SW biosynthesis. Six differentially expressed genes (DEGs) closely associated with SW synthesis were identified by transcriptomic analysis of *A. oxytropis* OW 7.8 and Δ*swnR*, with *P5CR*, *swnR*, *swnK*, *swnH2*, and *swnH1* downregulating, and *sac* upregulating. The expression levels of the six genes were consistent with the transcriptomic analysis results. Five differential metabolites (DEMs) closely associated with SW synthesis were identified by metabolomic analysis, with *L*-Lys, *L*-Glutamic acid, Saccharopine, and *L*-Proline upregulating, and *L*-PA downregulating. The results lay the foundation for the in-depth elucidation of molecular mechanisms and SW synthesis pathways in fungi, and are also of importance for the prevention of locoism in livestock, the control and utilization of locoweeds, and the protection and sustainable development of grassland ecosystems.

## 1. Introduction

Locoweed refers collectively to toxic plants of the genera *Oxytropis* and *Astragalus* that contain swainsonine (SW), representing one of the most detrimental poisonous plants to global animal husbandry [1,2]. Due to their strong stress tolerance and reproductive capacity, locoweeds exhibit superior competitive advantages in grassland ecosystems. These toxic plants are widely distributed across various regions worldwide, including the United States, Russia, Australia, Egypt, Mexico, Spain, Mongolia, and Iceland [3,4,5,6]. In China, locoweeds are predominantly found in northwestern areas such as Western Inner Mongolia, Ningxia, Gansu, Qinghai, and Xinjiang [3,7,8]. The consumption of locoweeds by livestock induces progressive intoxication characterized by weight loss, addictive grazing behavior, neurological dysfunction, and abortion in pregnant females, with severe cases culminating in mortality, thereby inflicting substantial economic losses on grassland livestock production systems [9,10,11,12,13].

The toxic alkaloid swainsonine (SW) was first isolated from *A. lentiginosus* and *O. sericea*, marking the first definitive identification of the causative toxin in locoweed poisoning [14]. Subsequent studies identified SW-producing endophytic fungi in *O. sericea*, *A. mollisimus*, and *O. lambertii*. Through combined ITS sequencing and morphological analyses, these fungi were initially classified as *Embellisia* species [15], later refined to the genus *Undifilum* [16], and ultimately revised to *Alternaria* species [17]. In addition to *A. oxytropis*, three other locoweed-associated endophytic fungi have been identified: *A. cinereum*, *A. fulva*, and *A. bornmuellerii* [16,17,18]. Notably, SW production has also been documented in various plant pathogens, insect pathogens, and human dermatopathogens [19,20,21]. SW is a fungal alkaloid toxin produced through secondary metabolism. Currently, the biosynthetic mechanisms of several mycotoxins, such as aflatoxins, deoxynivalenol (DON), and zearalenone (ZEN), have been well characterized, all being regulated by highly conserved gene clusters [22,23,24,25].

Our research group first isolated an SW-producing endophytic fungus from *O. glabra*. In vitro culture experiments confirmed SW biosynthesis by this fungal strain, while SW was undetectable in plants lacking this endophyte. These findings established that SW toxicity in *O. glabra* originates from its endophytic fungus. Through comprehensive characterization, we identified this organism as *Alternaria oxytropis* and designated it as *Alternaria oxytropis* OW7.8 [26,27,28].

The *sac* gene (*saccharopine reductase* gene) was knocked out in *A. oxytropis* OW7.8 by our research group previously, and the *sac* gene knockout mutant M1 was successfully screened out. The SW level of M1 cultured from 14 to 45 days was always lower than *A. oxytropis* OW7.8. The level of Saccharopine in M1 was lower than that of *A. oxytropis* OW7.8 [29]. The SW level of the *sac* gene complementary strain C1 was higher than that of *A. oxytropis* OW7.8 and M1, indicating that *sac* gene function promoted SW synthesis in *A. oxytropis* OW7.8 [30].

A comparative genomic analysis was conducted on diverse SW-producing fungi (*Metarhizium robertsii*, *Ipomoea carnea endophyte*, *Arthroderma otea*, *Trichophyton equinum*, *A. oxytropis*, and *Pseudo*-*gymnoascus* sp.), suggesting a conserved homologous gene cluster "SWN" comprising seven genes (*swnA*, *swnT*, *swnR*, *swnK*, *swnN*, *swnH2*, and *swnH1*) that are functionally associated with SW biosynthesis, with the encoded proteins and their respective functions detailed in Table 1 [31]. However, different fungi that produce SW do not necessarily all contain these seven genes. For example, there are no *swnA* and *swnT* genes in *A. oxytropis* and *Pyrenophora semeniperda*. *Slafractonia leguminicola* lacks the *swnA* gene, and *Chaetothyriaceae* sp. does not have the *swnT* gene [32].

Both *swnR* and *swnN* encode different reductases of the Rossmann-fold family and participate in the reduction reaction of two imine ions during SW synthesis. During the formation of 1-Hydroxyindolizine from 1-Oxoindolizine, the SwnN enzyme or SwnR enzyme catalyzed C3=N+4 imine ion to form 1-Hydroxyindolizine and the SwnN enzyme or SwnR enzyme catalyzed the formation of SW by C9=N+4 imine ion during the SW formation process of 1-Hydroxyindolizine [31]. The *swnR* gene, *swnN* gene, and *swnR* and *swnN* genes were knocked out in *M. robertsii*, and it was found that the SW level in each strain was Δ*swnNR* < Δ*swnN* < Δ*swnR* < WT; PA levels were significantly reduced in Δ*swnR* and Δ*swnN*, and extremely significantly decreased in Δ*swnNR*, suggesting that the SwnR enzyme in *M. robertsii* reduced P6C to PA [33].

The *swnN* gene of *A. oxytropis* OW7.8 was knocked out by our research group; however, SW was not detected in the mycelia of Δ*swnN* knockout strain cultured for 20 days, and the morphology and structure of Δ*swnN* colony and mycelia were changed and the growth rate was slower than that of *A. oxytropis* OW7.8. SW was detected in the mycelia of Δ*swnN*/*swnN*, a functional complementary strain of the *swnN* gene growing for 20 days, indicating that the *swnN* gene promoted SW synthesis [34]. Later, the *swnH1* gene of *A. oxytropis* OW7.8 was knocked out, and SW was not detected in Δ*swnH1* mycelia. The results showed that *swnH1* gene function also promoted SW biosynthesis [35].

**Figure 1:**
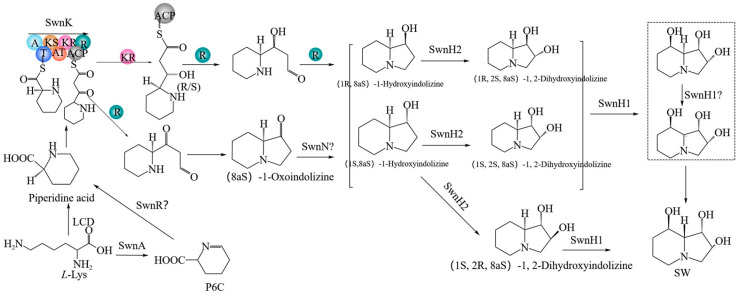
SW synthesis pathway in *M. robertsii* [33].

The SW synthesis pathway was first studied in *Rhizoctonia leguminicola* [36] and later in *M. robertsii* and *A. oxytropis* [31,33,37,38,39]. The SW biosynthetic pathway predicted in *R. Liguminicola* was [36] *L*-lysine → Saccharopine → α-Aminoadipic semialdehyde → P6C → PA → 1-Oxoindolizine → 1-Hydroxyindolizine → 1, 2-Dihydroxyindolizine → SW. At present, the predicted SW synthesis pathway in *M. robertsii* is relatively clear (Figure 1) [33]: *L*-Lys can be directly catalyzed by lysine cyclodeaminase (LCD) to synthesize PA, or *L*-Lys can be synthesized into P6C by the SwnA enzyme, and then reduced to PA by the SwnR enzyme. PA is catalyzed by non-ribopeptide-polyketo synthase (SwnK enzyme) to synthesize 1-Oxoindolizine or 1-Hydroxyindolizine. 1-Oxoindolizine is catalyzed by the SwnN enzyme to form 1-Hydroxyindolizine, followed by 1, 2-Dihydroxyindolizine catalyzed by the SwnH2 enzyme. Finally, 1, 2-Dihydroxyindolizine formed SW under the action of SwnH1.

**Figure 2:**
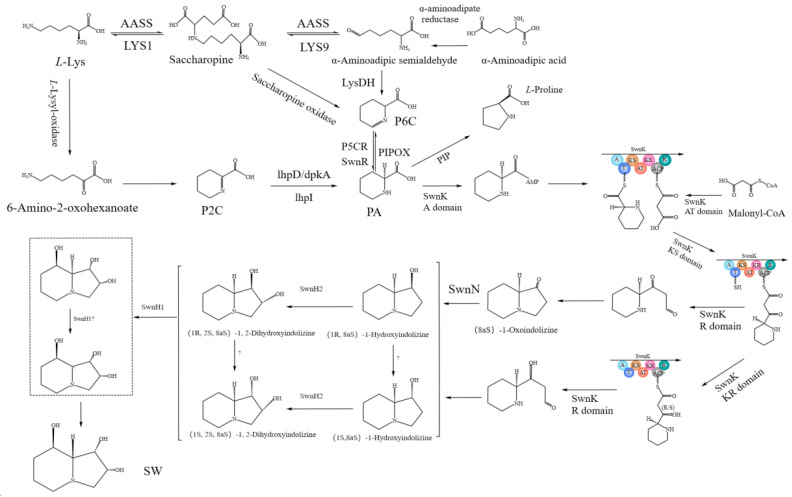
SW synthesis pathway in *A. oxytropis* OW 7.8 [34].

Based on the results of previous studies, our research group speculated the biosynthetic pathway of SW in *A. oxytropis* OW7.8 (Figure 2) [34], starting from *L*-lysine artificially. The Sac enzyme catalyzed the synthesis of saccharopine from *L*-Lysine. Saccharopine was reduced to α-aminoadipic semialdehyde (α-aminoadipic semialdehyde was also formed from α-aminoadipic acid catalyzed by α-aminoadipate reductase). α-Aminoadipic semialdehyde was catalyzed by LysDN to form P6C (P6C was also formed from saccharopine catalyzed by saccharopine oxidase). P6C was then catalyzed by the P5CR enzyme or SwnR enzyme to form *L*-PA. There might be an alternative pathway synthesizing *L*-PA, in which *L*-lysine was converted to 6-amino-2-oxohexanoate catalyzed by *L*-lysyl-alpha-oxidase. 6-Amino-2-oxohexanoate cyclized to form P2C, which was subsequently catalyzed by the enzymes lhpD/dpkA/lhpI to produce *L*-PA.

*L*-PA was converted to (8aS)-1-Oxoindolizine, (1R,8aS)-1-Hydroxyindolizine, or (1S,8aS)-1-Hydroxyindolizine by the action of multifunctional SwnK proteins. Subse-quently, (8aS)-1-Oxoindolizine was synthesized from (8aS)-1-Hydroxyindolizine by the SwnN enzyme. (1R,2S,8aS)-1,2-Dihydroxyindolizine was synthesized from (1R,8aS)-1-Hydroxyindolizine by the action of the SwnH2 enzyme, and (1S,2S,8aS)-1,2-Dihydroxyindolizine was synthesized from (1S,8aS)-1-Hydroxyindolizine catalyzed by the SwnH2 enzyme. Finally, (1S,2S,8R,8aR)-1,2,8-octahydroindolizinetriol (SW) was synthesized from (1R,2S,8aS)-1,2-Dihydroxyindolizine or (1S,2S,8aS)-1,2-Dihydroxyindolizine catalyzed by the SwnH1 enzyme.

In this study, the *swnR* gene of the endophytic fungus *Alternaria oxytropis* OW7.8 was cloned for the first time, the Δ*swnR* knockout strain and overexpressed strain *swnR*-OE were constructed, the SW levels in mycelia of each strain were detected, and the effects of the gene function on SW synthesis were studied. Additionally, transcriptome sequencing and metabolomic analysis were performed on *A. oxytropis* OW7.8 and the Δ*swnR* mutant. The findings provide crucial foundational data for elucidating the molecular mechanisms and metabolic pathways underlying fungal SW biosynthesis, which also hold significant implications for the management and utilization of locoweed.

## 2. Materials and Methods

### 2.1. Strain Cultivation

Solid culture: The *A. oxytropis* OW7.8 [27] (previously isolated from *O. glabra* in Wushen Banner, Ordos city, Inner Mongolia, China (108°52′ E, 38°36′ N, elevation 1291 m), hereafter referred to as OW7.8) was inoculated onto potato dextrose agar (PDA) media and incubated at 25 °C. Liquid culture: OW7.8 was inoculated into potato dextrose broth (PDB) and incubated at 25 °C with shaking.

### 2.2. Genomic DNA Extraction and swnR Gene Cloning from A. oxytropis OW7.8

The Genomic DNA was extracted from OW7.8 (Ezup Column Fungi Genomic DNA Purification Kit Tiangen, Beijing, China). The *swnR* gene was amplified using primers RF/RR (5′-TCCTGCGGAGCAATCTACCT-3′ and 5′-GTTATCCACTGGGAAGCCTCT-3′) under the following PCR conditions: initial denaturation at 94 °C for 3 min; 30 cycles of 94 °C for 30 s, 58 °C for 30 s, and 72 °C for 90 s; final extension at 72 °C for 10 min; and hold at 4 °C. The PCR products were detected by 1% agarose gel electrophoresis, purified (SanPrep Column PCR Product Purification Kit, Sangon Biotech, Shanghai, China), and then sequenced (Sangon Biotech, Shanghai, China).

### 2.3. Total RNA Extraction and swnR cDNA Cloning from A. oxytropis OW7.8

Total RNA of *A. oxytropis* OW 7.8 was extracted (OminiPlant RNA Kit, CWBIO, Shanghai, China) and reverse-transcribed to synthesize cDNA. The *swnN* cDNA was amplified with primers RcF/RcR (5′-CGCGGATCCATGCGTGTCGCGATCGCT-3′;5′-CGGCCCGGGTCAGATTATTGAATTGGGATA-3′). The PCR program was as follows: 94 °C for 3 min; 94 °C for 30 s, 58 °C for 30 s, 72 °C for 60 s, repeated for 30 cycles; and a final extension at 72 °C for 10 min. The PCR products were detected by 1.0% agarose gel electrophoresis, purified (SanPrep Column PCR Product Purification Kit, Sangon Biotech, Shanghai, China), and then sequenced (Sangon Biotech, Shanghai, China).

### 2.4. Vectors Construction

#### 2.4.1. Construction of the swnR Gene Knockout Vector

The upstream and downstream homologous regions of the *swnR* gene were amplified from *A. oxytropis* OW7.8 genomic DNA using primer pairs RsF/RsR (5′-CGCGGATCCCAGGCAAAGAGTTCCAAATCG-3′; 5′-GACCTCCACTAGCTCCAGCCAAGCCCATCGCATCTCGCTTCGTGA-3′) and RxF/RxR (5′-ATAGAGTAGATGCCGACCGCGGGTTCATATCCAGGCATTGCACTCAGC-3′; 5′-CGGAATTCGACTTGATTCGTGCGGTGTAA-3′). The 5′ and 3′ regions of the *hpt* gene were amplified from plasmid pCB1003 using primers hphsF/hphsR (5′-TCACGAAGCGAGATGCGATGGGCTTGGCTGGAGCTAGTGGAGGTC-3′; 5′-GTATTGACCGATTCCTTGCGGTCCGAA-3′) and hphxF/hphxR (5′-GATGTAGGAGGGCGTGGATATGTCCT-3′; 5′-GCTGAGTGCAATGCCTGGATATGAACCCGCGGTCGGCATCTACTCTAT-3′). The *swnR* knockout cassette (432 bp upstream homologous region + 1380 bp hygromycin phosphotransferase gene + 449 bp downstream homologous region) was assembled using split-marker recombination and subsequently cloned into the pMD-19-T Vector (Takara, Beijing, China) via TA cloning.

#### 2.4.2. Construction of the swnR Gene Overexpression Vector

The plasmid pBARGPE1-Hygro and the purified *swnR* cDNA fragment were digested with restriction enzymes *BamH*I and *Xma*I. The digested products were purified and ligated overnight at 16 °C using T4 DNA ligase.

### 2.5. Hygromycin Sensitivity Assay of A. oxytropis OW7.8

The mycelia of *A. oxytropis* OW7.8 was cultured on PDA media supplemented with Hyg B at concentrations of 0 μg/mL, 0.5 μg/mL, 0.6 μg/mL, 0.7 μg/mL, 0.8 μg/mL, 0.9 μg/mL, 1 μg/mL, and 2 μg/mL. Colony growth was observed to determine the minimum Hyg B concentration required to inhibit OW7.8 growth, which was subsequently used for selecting knockout transformants.

### 2.6. Protoplast Preparation and Transformation of A. oxytropis OW7.8

Young mycelia of OW7.8 were inoculated in PDB media (50 μg/mL Amp) shaking at 25 °C, 200 rpm for 7 days. Protoplasts from mycelia of *A. oxytropis* OW 7.8 were prepared according to the method described by Hu et al. [40]. The *swnR* gene knockout vector was then transformed into protoplast of *A. oxytropis* OW 7.8 mediated by PEG8000.

### 2.7. Screening and Verification of Gene Knockout Transformants

The *swnR* gene transformants were cultured on TB_3_ regeneration media (base layer: 50 μg/mL ampicillin and 1 μg/mL hygromycin B; top layer: 50 μg/mL Amp and 2 μg/mL Hyg B) at 25 °C until single colonies appeared, which were then transferred to PDA media (2 μg/mL Hyg B) for continuous cultivation until stable growth was achieved. Genomic DNA was extracted from the transformants and subjected to PCR amplification using three primer sets: hptF/hptR (5′-GGCTTGGCTGGAGCTAGTGGAGGTCAA-3′/5′-GAACCCGCGGTCGGCATCTACTCTAT-3′) for *hpt* gene sequence verification, RsF/hptsR (5′-CGCGGATCCCAGGCAAAGAGTTCCAAATCG-3′/5′-GTATTGACCGATTCCTTGCGGTCCGAA-3′) for amplification of the upstream homologous arm + *hpt* gene sequence, and hptxF/RxR (5′-GATGTAGGAGGGCGTGGATATGTCCT-3′/5′-CGGAATTCGACTTGATTCGTGCGGTGTAA-3′) for the *hpt* gene sequence combined with downstream homologous arm, with all PCR products subsequently sequenced for confirmation. Additionally, primers nRF/nRR were used to amplify the internal sequence of the *swnR* gene using both OW7.8 and the putative knockout transformants as templates. The successfully verified *swnR* gene knockout mutant of *A. oxytropis* OW7.8 was formally designated as Δ*swnR*.

### 2.8. Transformation of A. oxytropis OW7.8 Protoplasts with swnR Overexpression Vector

Young mycelia of OW7.8 were inoculated in PDB media (50 μg/mL Amp) shaking at 25 °C, 200 rpm for 7 days. Protoplasts from mycelia of *A. oxytropis* OW 7.8 were prepared according to the method described by Hu et al. [40]. The *swnR* overexpression vector was then transformed into protoplast of *A. oxytropis* OW 7.8 mediated by PEG8000.

### 2.9. Screening and Verification of Overexpression Transformants

The *swnR*-overexpressing transformants were cultured on TB_3_ regeneration media (base layer: 50 μg/mL ampicillin and 1 μg/mL hygromycin B; top layer: 50 μg/mL ampicillin and 2 μg/mL hygromycin B) at 25 °C until single colonies emerged, which were subsequently transferred to PDA media (50 μg/mL ampicillin and 2 μg/mL hygromycin B) for continuous cultivation until stabilization. Genomic DNA was extracted from the putative overexpression transformants, followed by PCR amplification using primer pairs hphF/hphR (5′-GGCTTGGCTGGAGCTAGTGGAGGTCAA-3′/5′-GAACCCGCGGTCGGCATCTACTCTAT-3′) for the hpt resistance gene and ROE-F/ROE-R (5′-CTAGAGGATCCATGCGTGTCG-3′/5′-CTTGATATCGAATTCCTGCAGCC-3′) for *swnR* cDNA verification. The successfully identified *swnR*-overexpressing strains were designated as *swnR*-OE.

### 2.10. Colony and Hyphal Morphology

The colony morphology of *A. oxytropis* OW 7.8, Δ*swnR*, and *swnR*-OE after 20 days of cultivation was observed. The mycelia morphology of *A. oxytropis* OW 7.8 and Δ*swnR* was observed under a scanning electron microscope (SU81003.0 kV × 30, Wuhan Servicebio Technology, Wuhan, China).

### 2.11. Extraction and Detection of SW in Mycelia of A. oxytropis OW 7.8, ΔswnR, and swnR-OE Mycelia

Mycelia of *A. oxytropis* OW 7.8, Δ*swnR*, and *swnR*-OE cultured for 20 days were used for SW extraction by acetic acid–chloroform solution, purification by cation exchange resin, and elution with 1 mol/L ammonia solution. The SW levels in the mycelia were determined by HPLC-MS, with three replicates for each sample. Data were analyzed by one-way ANOVA in GraphPad Prism 9.5 software. The chromatographic conditions were as follows: mobile phase, 5% methanol and 20 mmol/L ammonium acetate; flow rate, 0.4 mL/min; column temperature, 30 °C. MS conditions were as follows: positive ion 156; negative ion 70; IonSpray voltage (IS), 30 V.

### 2.12. Transcriptomic and Metabolomic Sample Processing and Data Analysis

Mycelia of *A. oxytropis* OW7.8 and Δ*swnR* were cultured on PDA at 25 °C for 20 days. Single colonies (0.1–0.3 g fresh weight) were collected and flash-frozen in liquid nitrogen (1–3 min) with three biological replicates. Transcriptome sequencing was performed on the Illumina NovaSeq 6000 platform (Novogene, Beijing, China). Data quality was assessed using Agilent 2100 bioanalyzer and Qubit 2.0 Fluorometer. HISAT2 and featureCounts were used for alignment and quantification, with DESeq2 identifying differentially expressed genes (DEGs) (criteria: |log_2_(Fold Change)| > 1 and padj < 0.05). DEGs were annotated using GO (http://www.geneontology.org, accessed on 25 February 2025.) and KEGG (https://www.genome.jp/kegg/pathway, accessed on 25 February 2025.) databases [41,42,43].

Metabolomic samples were processed identically to transcriptomic samples, with five biological replicates. HPLC-MS/MS analysis was performed. Data quality was evaluated using orthogonal partial least squares-discriminant analysis (OPLS-DA) and variable importance in projection (VIP) scores. The metaX software 2.0 processed metabolomic data. Differential metabolites (VIP > 1, |log_2_(Fold Change)| > 1, and padj < 0.05) were identified using Python (Python-3.5.0, Python-2.7.6) and R (R-3.4.3, R-4.0.3), with KEGG database annotation [44,45,46].

### 2.13. RT-qPCR Analysis of SW Biosynthesis-Associated Genes in OW7.8, ΔswnR, and swnR-OE

RT-qPCR was performed using cDNA templates derived from OW7.8 and Δ*swnR*, with the actin gene serving as the endogenous reference. Gene-specific amplification was conducted using the following primer pairs: sacQF/sacQR (5′-GTCAACGATGCCGATGCTCTC-3′/5′-TGCGGATTGCGGACTTGATAAC-3′), P5CRQF/P5CRQR (5′-CAATAATGGGCGGCGTGATGTC-3′/5′-ACAGGCGATGAAGTTGGAGAGG-3′), swnRQF/swnRQR (5′-TTCTACTTTGCCACACACGAACC-3′/5′-GTCAGCCAACCAGCCAATGC-3′), swnKQF/swnKQR (5′-CTTGCTCGCCTGTGCTGATTC-3′/5′-CGTCAACTCGTCCAACACTTCC-3′), swnNQF/swnNQR (5′-ATGCCATTGCGGAATCAGGTAAG-3′/5′-CGTCTTGTTGGTTGCGGTAGTAG-3′), swnH2QF/swnH2QR (5′-CATCTGCTCCTCGCTTGCTACC-3′/5′-CAGGACAACGCCTCCATCTCTTTC-3′), and swnH1QF/swnH1QR (5′-TTTGCTTTGCGGAGATGGAACC-3′/5′-CTGGACGGAGTGTGCCTGAG-3′). The PCR program was as follows: 95 °C for 30 s; 95 °C for 5 s, 60 °C for 30 s, repeated for 29 cycles, 95 °C for 5 s, 65 °C for 1 min, 95 °C for 20 s. All experimental samples were analyzed in triplicate, with relative gene expression levels (*sac*, *P5CR*, *swnR*, *swnK*, *swnN*, *swnH1*, and *swnH2*) calculated using the 2^−ΔΔCt^ method, where ΔCt = Ct (target gene) − Ct(actin) and ΔΔCt = ΔCt (∆*swnR*) − ΔCt (OW7.8). Statistical analysis was performed using GraphPad Prism 9.5.0 (one-way ANOVA with post hoc testing).

## 3. Results

### 3.1. Cloning and Bioinformatics Analysis of swnR Gene in A. oxytropis OW7.8

**Figure 3:**
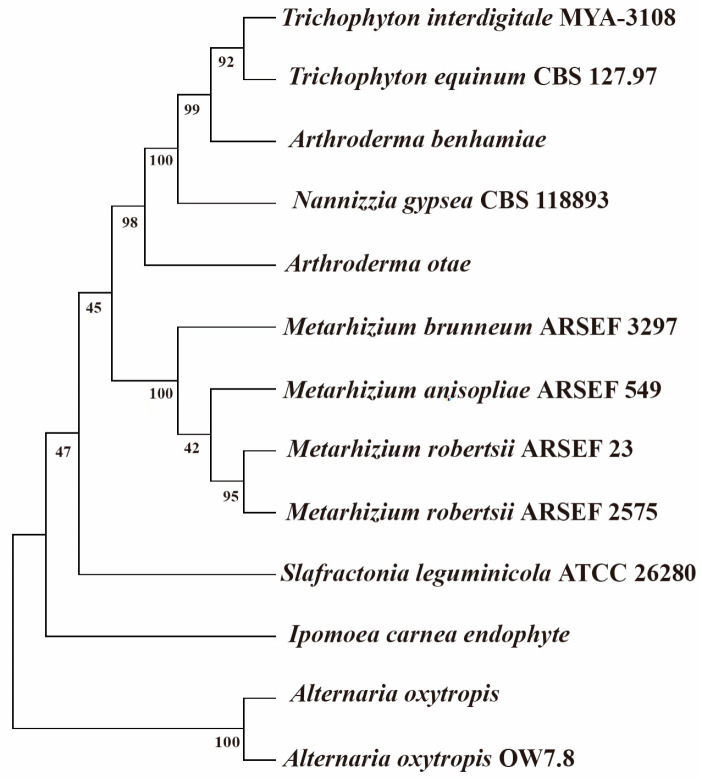
Neighbor-joining phylogenetic tree of SwnR amino acid sequences.

The *swnR* gene (GenBank: OP834145) was successfully cloned from the locoweed endophytic fungus *A. oxytropis* OW7.8, comprising 918 bp (ATG-TGA) without introns and encoding a predicted 305-amino acid protein with a molecular weight of 34.87 kDa and pI of 6.15. Bioinformatics analysis predicted the encoded SwnR protein to be non-transmembrane and hydrophilic. Phylogenetic analysis of SwnR amino acid sequences from 12 SWN gene cluster-containing fungi (Figure 3) revealed 100% identity with *Alternaria oxytropis* (AQV04226.1), while showing 72.13% identity with *Slafractonia leguminicola*, 69.81% with *Ipomoea carnea* endophyte (KY365740), 69.51% with both *Metarhizium brunneum* ARSEF 3297 and *Metarhizium robertsii* (ARSEF 23 and ARSEF 2575), and 69.18% with *Metarhizium anisopliae* ARSEF 549.

### 3.2. Hygromycin B Sensitivity of A. oxytropis OW7.8

**Figure 4:**
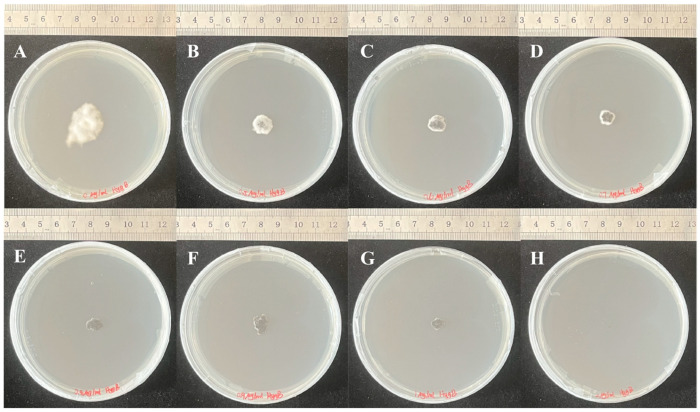
Colony morphology of *A. oxytropis* OW7.8 following 30-day cultivation on PDA media containing different Hyg B concentrations. Note: (**A**): 0 μg/mL, (**B**): 0.5 μg/mL, (**C**): 0.6 μg/mL, (**D**): 0.7 μg/mL, (**E**): 0.8 μg/mL, (**F**): 0.9 μg/mL, (**G**): 1 μg/mL, (**H**): 2 μg/mL.

The growth characteristics of OW7.8 colonies cultured for 30 days on PDA media containing varying Hyg B concentrations (0, 0.5, 0.6, 0.7, 0.8, 0.9, 1, and 2 μg/mL) are presented in Figure 4. The results demonstrated complete growth inhibition of *A. oxytropis* OW7.8 at Hyg B concentrations ≥ 2 μg/mL in PDA media, establishing this concentration as the threshold for effective selection pressure.

### 3.3. Screening and Verification of *Δ*swnR Mutants

**Figure 5:**
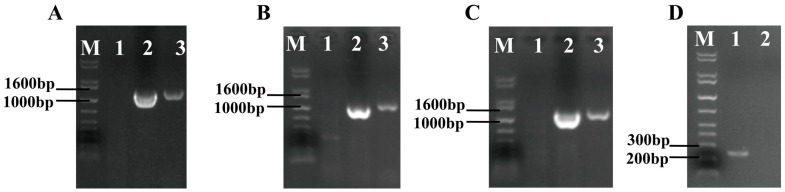
Electrophoretic analysis of PCR products from Δ*swnR* genomic DNA. M: 1 kb Plus DNA Ladder. (**A**): Lane 1: Negative control; Lane 2: Positive control; Lane 3 shows a band of the *hpt* gene (expected 1388 bp). (**B**): Lane 1: Negative control; Lane 2: Positive control; Lane 3 shows a band of the *swnR* upstream homologous sequence + *hpt* gene (expected 1205 bp). (**C**): Lane 1: Negative control; Lane 2: Positive control; Lane 3 shows a band of the *swnR* downstream homologous sequence + *hpt* gene (expected 1388 bp). (**D**): Lane 1: Positive control (*swnR* internal sequence, expected 261 bp); Lane 2: PCR product using Δ*swnR* genomic DNA as template.

PCR analysis confirmed successful genomic integration in the *swnR* knockout transformants, demonstrating amplification products for the *hpt* resistance gene, the upstream homologous sequence of the *swnR* and *hpt* genes, and the *hpt* gene and the downstream homologous sequence of *swnR* (Figure 5), while failing to amplify the internal *swnR* sequence. The sequencing of the PCR products confirmed their accuracy, resulting in the identification of the ∆*swnR*.

### 3.4. Screening and Identification of swnR-OE Transformants

**Figure 6:**
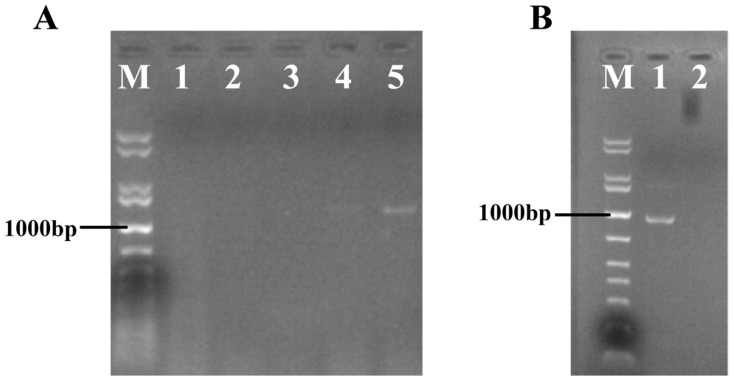
Electrophoretic analysis of PCR products from *swnR*-overexpressing transformant. M: 1 kb plus DNA ladder. (**A**) Lane 1–3: Negative control; Lanes 4,5: *hpt* gene fragment (expected 1380 bp). (**B**) Lane 1: Negative control; Lanes 2: *swnR* gene sequence (expected 956 bp).

A PCR analysis of the putative swnR-overexpressing transformants successfully amplified both the *hpt* gene and the *swnR* gene sequence (Figure 6), with subsequent sequencing confirming the correct amplification of the expected fragments.

### 3.5. Colony and Hyphal Morphology

**Figure 7:**
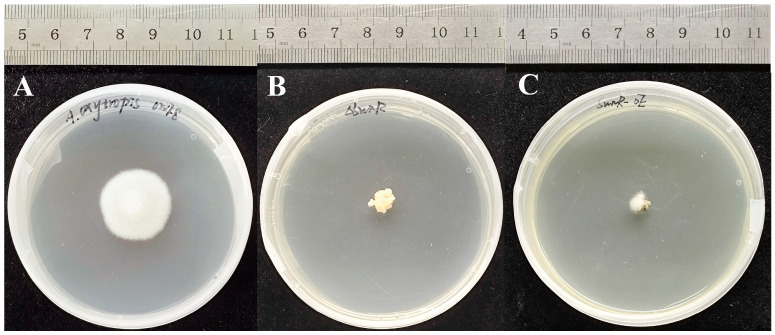
Comparison of *A. oxytropis* OW7.8, Δ*swnR*, and *swnR*-OE colony morphology. Note: (**A**): *A. oxytropis* OW7.8; (**B**): Δ*swnR*; (**C**): *swnR*-OE.

The *A. oxytropis* OW7.8 exhibited circular colonies with central protrusions, characterized by white fluffy mycelia forming loosely arranged, radially distributed hyphae and distinct melanin pigmentation on the reverse side. In contrast, the Δ*swnR* knockout mutant displayed raised, cream-to-pale yellow colonies with irregular margins, featuring short, swollen-tip hyphae in granular arrangements without pigment accumulation, demonstrating slower growth rates and stratified colony architecture compared with the wild-type. The *swnR*-OE developed irregularly raised colonies with reduced mycelial density but retained reverse-side pigmentation, though exhibiting slower growth than OW7.8 (Figure 7).

**Figure 8:**
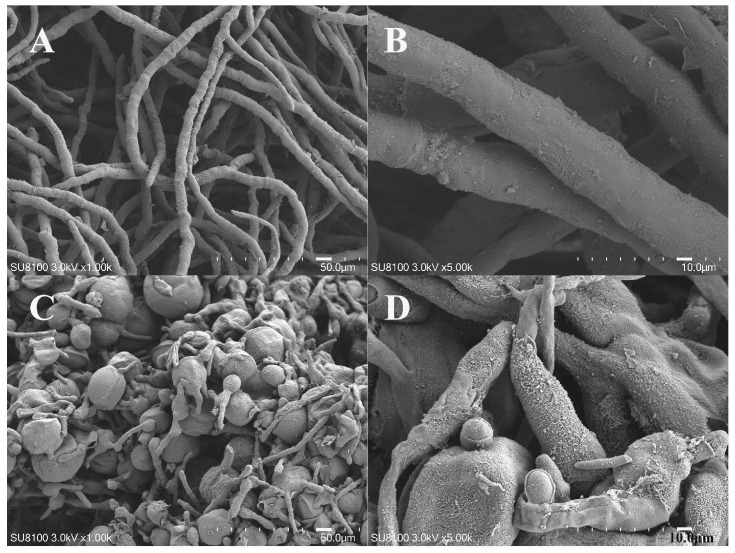
Comparative scanning electron micrographs of OW7.8 and Δ*swnR* hyphal morphology. Note: (**A**): *A.oxytropis* OW 7.8 mycelia magnified 1000×, (**B**): *A.oxytropis* OW 7.8 mycelia magnified 5000×, (**C**): Δ*swnR* mycelia magnified 1000×, (**D**): Δ*swnR* mycelia magnified 5000×.

The *A. oxytropis* OW7.8 exhibited typical fungal hyphal morphology, characterized by slender, well-developed hyphae with clearly visible septa, whereas the Δ*swnR* mutant displayed significantly altered mycelial architecture featuring shortened hyphae with bulbous tips and an overall densely packed structure, as illustrated in Figure 8.

### 3.6. SW Levels in A. oxytropis OW 7.8, *Δ*swnR and swnR-OE Mycelia

**Figure 9:**
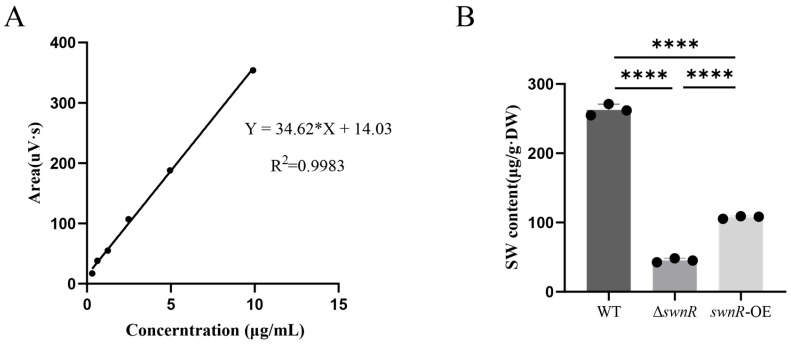
SW level detection in OW7.8, Δ*swnR*, and *swnR*-OE in 20 d. (**A**): SW standard curve. (**B**): SW content in OW7.8, Δ*swnR*, and *swnR*-OE in 20th d. The error bar represents the standard error of the mean (n = 3), with (****) *p* < 0.0001.

The standard curve for SW quantification (Figure 9A) was established by plotting peak areas against known SW concentrations, yielding a linear regression equation Y = 34.62 X + 14.03 (R^2^ = 0.9983). After 20 days of cultivation, HPLC analysis revealed significantly different SW levels among the strains: wild-type OW7.8 accumulated 262.6569 ± 8.1911 μg/g DW, while the Δ*swnR* knockout mutant showed markedly reduced production (45.3986 ± 12.8587 μg/g DW). The *swnR*-OE exhibited intermediate SW levels (108.4023 ± 2.7297 μg/g DW), demonstrating a significant increase compared with Δ*swnR* but not reaching wild-type concentrations, confirming the functional role of the *swnR* gene in promoting SW biosynthesis in *A. oxytropis* OW7.8 (Figure 9B).

### 3.7. Transcriptome Sequencing Analysis of A. oxytropis OW7.8 and *Δ*swnR

**Figure 10:**
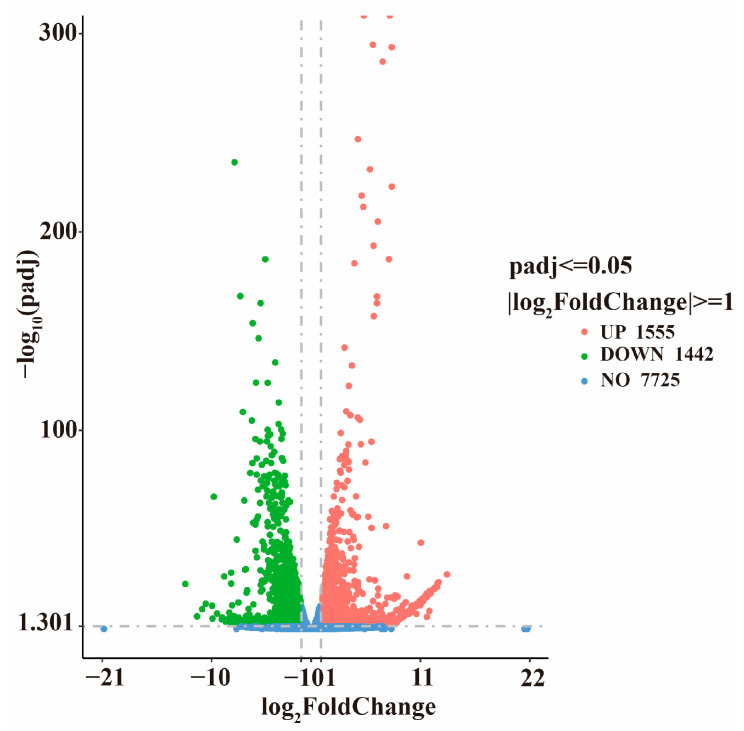
Volcano map of *A. oxytropis* OW7.8 and Δ*swnR* DEGs. Note: x: log_2_FoldChange value, y: −log_10_ (padj). Red represents upregulated genes, green represents downregulated genes, and blue represents genes with no significant differential expression.

Transcriptome sequencing of *A. oxytropis* OW7.8 and Δ*swnR* generated a total of 288,360,652 clean reads, amounting to 43.26 G of data. The results revealed significant differential gene expression between *A. oxytropis* OW7.8 and Δ*swnR*. A total of 2997 differentially expressed genes (DEGs) were identified, of which 1555 (51.89% of DEGs) were upregulated and 1442 (48.11%) were downregulated. Among these, six DEGs closely associated with SW biosynthesis were identified, including the upregulated *sac* gene and the downregulated *P5CR*, *swnN*, *swnK*, *swnH1*, and *swnH2* genes (Figure 10).

**Figure 11:**
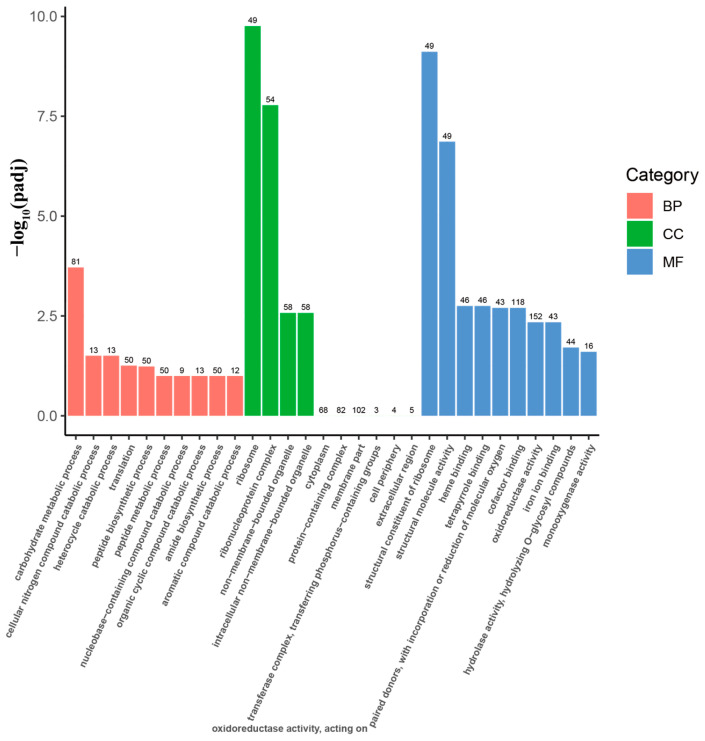
GO enrichment of DEGs in *A. oxytropis* OW 7.8 and ∆*swnR*. Note: BP: Biological Process, CC: Cellular Component, MF: Molecular Function.

The GO (Gene Ontology) enrichment analysis of differentially expressed genes (DEGs) between *A. oxytropis* OW7.8 and Δ*swnR* (Figure 11) identified 1018 annotated DEGs, comprising 546 upregulated and 472 downregulated genes, which were functionally categorized into 280 biological process (BP) terms, 70 cellular component (CC) terms, and 180 molecular function (MF) terms. Within BP categories, carbohydrate metabolic processes showed differential expression of 28 upregulated and 53 downregulated genes, while cellular nitrogen compound catabolic processes and heterocycle catabolic processes each exhibited seven upregulated and six downregulated genes. The CC analysis revealed predominant DEG enrichment in ribosome-related terms, with 49 genes upregulating in ribosome and 52 upregulating plus two downregulating genes in the ribonucleoprotein complex. For MF categories, significant enrichment was observed for the structural constituent of ribosome (49 upregulated genes) and structural molecule activity (49 upregulated genes), demonstrating substantial transcriptional reprogramming in cellular architecture-related functions.

**Figure 12:**
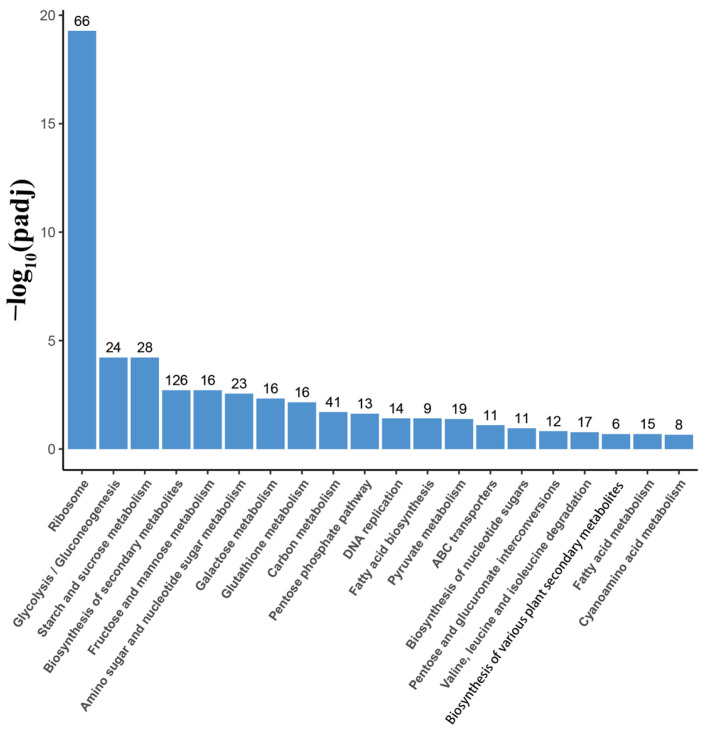
KEGG enrichment of DEGs in *A. oxytropis* OW 7.8 and ∆*swnR*.

The KEGG pathway analysis identified 411 differentially expressed genes (DEGs) between *A. oxytropis* OW7.8 and the Δ*swnR* (159 upregulated and 252 downregulated), which were significantly enriched in 99 metabolic pathways (Figure 12). Among the most prominently altered pathways (padj ≤ 0.05), ribosomal biosynthesis showed substantial upregulation with 66 genes, while carbohydrate metabolism pathways exhibited distinct expression patterns: glycolysis/gluconeogenesis displayed 2 upregulated and 22 downregulated genes, and starch/sucrose metabolism contained 7 upregulated versus 21 downregulated genes.

### 3.8. Metabolomic Profiling of A. oxytropis OW7.8 and *Δ*swnR

**Figure 13:**
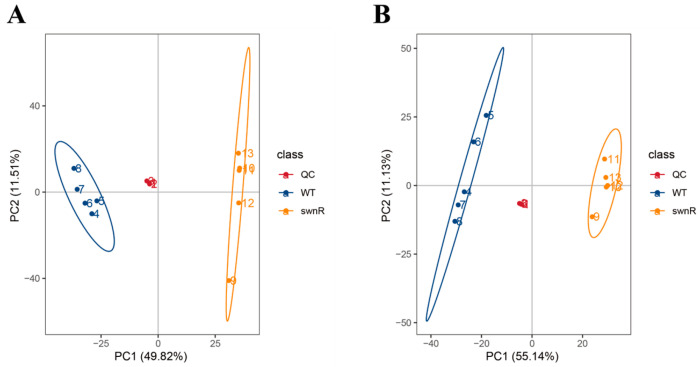
PCA analysis of total samples based on different model subsets. (**A**). Positive ion mode (**B**). Negative ion mode.

Principal component analysis (PCA) of the metabolomic data revealed distinct metabolite profiles between Δ*swnR* and *A. oxytropis* OW7.8 in both positive and negative ion modes (Figure 13).

**Figure 14:**
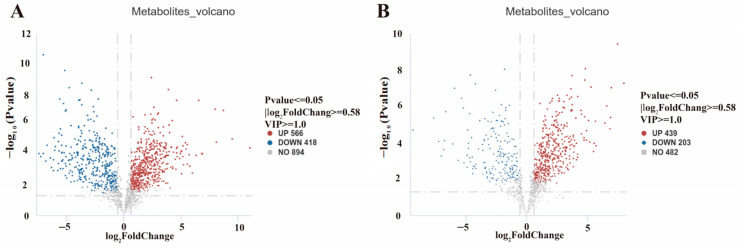
*A. oxytropis* OW 7.8 and Δ*swnR* differential metabolite volcano plot. (**A**). Positive ion mode (**B**). Negative ion mode.

Comprehensive analysis identified 1626 significantly differential metabolites (DEMs), with 1005 upregulated and 621 downregulated in Δ*swnR* compared with the wild-type strain. Specifically, positive ion mode detection yielded 984 DEMs (566 upregulated and 418 downregulated), while negative ion mode analysis identified 642 DEMs (439 upregulated and 203 downregulated), as detailed in Table 2 and Figure 14.

**Figure 15:**
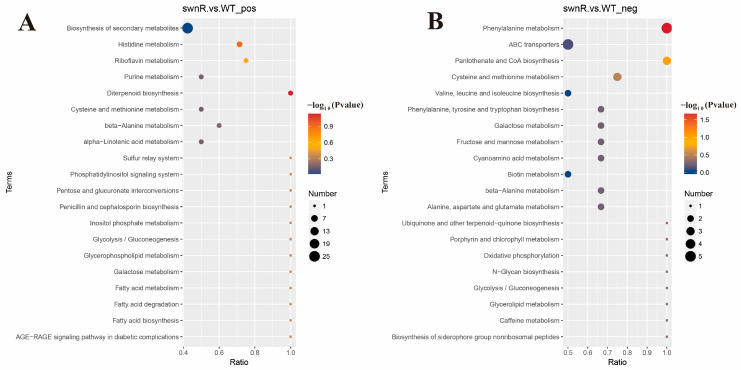
Bubble chart of KEGG enrichment analysis of differential metabolites between *A. oxytropis* OW 7.8 and Δ*swnR* strains in positive and negative ion modes. (**A**). Positive ion mode (**B**). Negative ion mode.

KEGG-based cluster analysis of differential metabolites revealed distinct metabolic patterns between *A. oxytropis* OW7.8 and Δ*swnR* (Figure 15), with the positive ion mode showing significant alterations in secondary metabolite biosynthesis and histidine metabolism pathways closely associated with SW production, while the negative ion mode demonstrated marked changes in phenylalanine metabolism and the alanine/aspartate/glutamate metabolic axis. Further screening identified five key differential metabolites linked to SW biosynthesis: *L*-lysine, *L*-glutamic acid, saccharopine, and *L*-proline were upregulated in Δ*swnR* compared with *A. oxytropis* OW7.8, whereas *L*-pipecolic acid showed significant downregulation.

### 3.9. Expression of sac, P5CR, and SWN Cluster Genes in A. oxytropis OW 7.8 and *Δ*swnR

**Figure 16:**
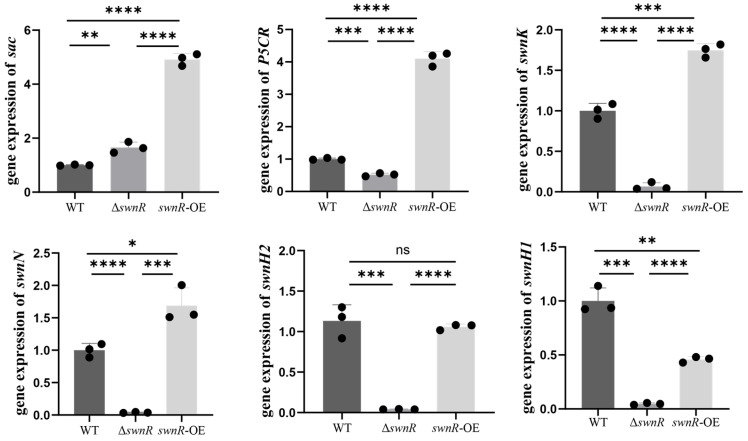
RT-qPCR detection of SW synthesis-related gene expression levels in *A. oxytropis* OW 7.8, Δ*swnR*, and *swnR*-OE. Note: The *x*-axis represents genes; the *y*-axis represents relative expression levels. Error bars indicate the standard error of the mean (n = 3), with (*) *p* < 0.05, (**) *p* < 0.01, (***) *p* < 0.001, (****) *p* < 0.0001.

Comparative RT-qPCR analysis revealed differential expression patterns of SW biosynthetic genes among the studied strains (Figure 16). In the Δ*swnR*, significant upregulation of the *sac* gene was observed alongside marked downregulation of the *P5CR*, *swnK*, *swnN*, *swnH2*, and *swnH1* genes compared with *A. oxytropis* OW7.8. The *swnR*-OE overexpression strain exhibited increased transcript levels of the *sac*, *P5CR*, *swnK*, and *swnN* gene, while showing decreased *swnH1* gene expression and unaltered *swnH2* gene expression levels.

**Figure 17:**
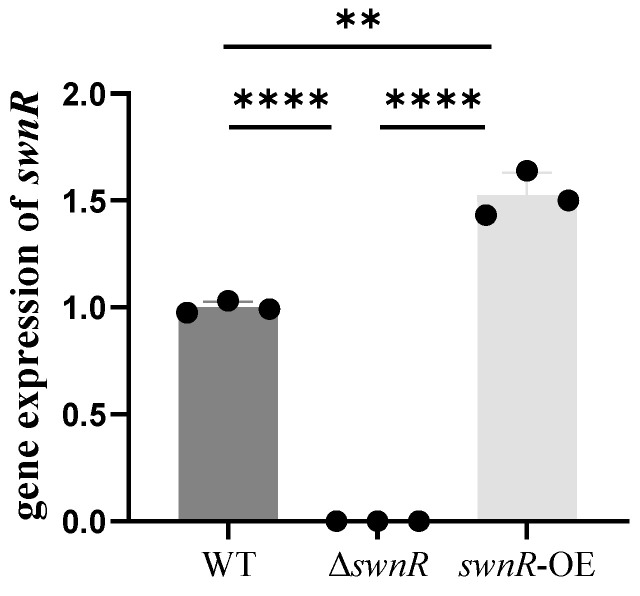
RT-qPCR detection of *swnR* gene expression levels in *A. oxytropis* OW 7.8, Δ*swnR*, and *swnR*-OE. Note: The *x*-axis represents genes; the *y*-axis represents relative expression levels. Error bars indicate the standard error of the mean (n = 3), with (**) *p* < 0.01, (****) *p* < 0.0001.

Furthermore, *swnR* gene expression analysis demonstrated a complete absence of transcription in Δ*swnR* and significant overexpression in *swnR*-OE relative to the wild-type strain (Figure 17).

## 4. Discussion

### 4.1. Functional Role of SwnR Reductase and swnR Gene in SW Biosynthesis in A. oxytropis OW7.8

The *swnR* gene (OP834145) encodes a reductase that catalyzes the conversion of P6C to PA in SW-producing fungi. Comparative sequence analysis revealed 100% amino acid identity between *A. oxytropis* OW7.8 and *A. oxytropis* Raft River (KY365741.1), with both sequences encoding 305 amino acids and lacking introns, indicating a close phylogenetic relationship [31]. The homologous gene in *M. robertsii* showed 69.51% sequence identity while maintaining a protein with 305 amino acids and an intronless structure.

Due to the inherent challenges of working with the slow-growing endophytic fungus *A. oxytropis* OW7.8, which exhibits extremely low homologous recombination efficiency during in vitro culture, extensive screening efforts yielded limited transformants—specifically, four knockout mutants and five overexpression strains after prolonged experimentation. SW production in Δ*swnR* mycelia decreased significantly, attributable to the loss of SwnR enzymatic activity required for PA biosynthesis from P6C, thereby limiting downstream SW formation. The *swnR*-OE strains showed higher SW levels than Δ*swnR*, and lower SW levels than OW7.8, confirming swnR’s positive role in SW biosynthesis. RT-qPCR analysis of *swnR*-OE revealed the coordinated upregulation of key SW pathway genes (*sac*, *P5CR*, *swnR*, *swnK*, and *swnN*), while the downregulation of the *swnH1* gene may explain the incomplete SW production recovery.

Previous whole-genome sequencing of *A. oxytropis* OW7.8 by our research group identified the *P5CR* gene, which encoded that the P5CR enzyme alternatively catalyzes P6C reduction to PA [47]. Consistent with our current findings, Δ*P5CR* similarly exhibited significantly reduced SW production compared with the wild-type. Molecular docking predictions revealed stronger binding affinity between SwnR and P6C (binding free energy: −4.56 kcal/mol) than the P5CR–P6C interaction (−4.0 kcal/mol), suggesting preferential P6C binding by SwnR.

These results align with studies in other fungal systems: Luo et al. demonstrated in *M. robertsii* that SW production followed Δ*swnNR* < Δ*swnN* < Δ*swnR* < WT, with parallel PA level measurements showing significant reduction in Δ*swnR* and extreme depletion in Δ*swnNR*, while Δ*swnN* accumulated PA, collectively supporting SwnR’s role in P6C → PA conversion [33]. Similarly, Sun et al. reported markedly decreased SW production in *M. anisopliae* Δ*swnR*, and that the SW level was partially restored in the gene function complementary strain relative to the wild-type [48]. Our conclusions regarding *swnR* gene function in SW biosynthesis thus showed cross-species consistency with these independent investigations in *Metarhizium* species.

### 4.2. Colony Morphology and SW Extraction and Purification

The *A. oxytropis* OW7.8 exhibited circular, white, and fluffy colonies with relatively dense hyphae, whereas the Δ*swnR* displayed pale yellow, loosely structured colonies with irregular margins, lacking pigment accumulation and showing reduced growth rates. These observations indicated that *swnR* gene knockout leads to abnormal hyphal morphology and impaired cellular proliferation. Electron microscopy further revealed shortened hyphae with swollen tips and an overall compact, stacked architecture. The *swnR*-OE formed irregularly raised white colonies with pigmented undersides and similarly exhibited slower growth.

For SW extraction from mycelia of *A. oxytropis* OW7.8, Δ*swnR*, and *swnR*-OE, freeze-dried hyphae were rapidly ground into a fine powder to minimize SW loss. Optimal amounts of glass fibers and cation-exchange resin were added, followed by elution with 1 mol/L ammonia, and the resulting SW-containing eluate was aliquoted into 1.5 mL centrifuge tubes while avoiding overfilling to prevent leakage during centrifugation, prior to drying using a rotary evaporator with limited exposure time. Samples intended for delayed SW quantification were sealed and stored at −20 °C to prevent moisture evaporation and ensure measurement accuracy.

### 4.3. Transcriptomic and Metabolomic Profiling of A. oxytropis OW7.8 and *Δ*swnR

Comparative transcriptomic analysis between *A. oxytropis* OW7.8 and the Δ*swnR* mutant revealed the significant downregulation of key SW biosynthetic genes including *P5CR*, *swnK*, *swnN*, *swnH2*, and *swnH1*, while the *sac* gene showed marked upregulation. Corresponding metabolomic profiling demonstrated elevated levels of *L*-Lys and saccharopine, consistent with the increased expression of the Sac enzyme responsible for saccharopine-to-*L*-lysine conversion. The knockout of the *swnR* gene resulted in reduced accumulation of both PA and SW, accompanied by the downregulation of the *P5CR* gene. Notably, the enzymatic reactions catalyzed by the *swnK*, *swnN*, *swnH2*, and *swnH1* genes all operate downstream of SwnR-mediated catalysis, and all four genes exhibited decreased expression patterns. These transcriptional changes were subsequently validated by RT-qPCR analysis, confirming the reliability of the transcriptome data. However, current transcriptomic platforms showed technical limitations in detecting several other potentially relevant genes, including *α-aminoadipate reductase*, *saccharopine oxidase*, L-lysyl-alpha-oxidase, *lysDH*, and *dpkA/lhpD/lhpI*, which were not successfully enriched in our analysis.

Metabolomic analysis identified five differentially accumulated metabolites closely associated with SW biosynthesis in ΔswnR. Notably, *L*-PA was significantly downregulated, while *L*-Lys, *L*-glutamic acid, *L*-proline, and saccharopine showed marked upregulation. Among these metabolites, *L*-Lys, saccharopine, and *L*-PA served as crucial precursors in the SW biosynthetic pathway. The observed reduction in *L*-PA levels likely results from the knockout of *swnR* gene, which abolished the SwnR enzyme activity required for the reduction of P6C to PA, though alternative biosynthetic routes may partially compensate for PA production. The accumulation of *L*-Lys could be attributed to increased saccharopine levels coupled with the upregulation of the *sac* gene, leading to enhanced conversion of saccharopine to *L*-Lys catalyzed by the Sac enzyme. Concurrent transcriptomic analysis revealed the elevated expression of the *proline iminopeptidase* gene, whose encoded enzyme may catalyze the conversion of PA to *L*-proline, thereby contributing to the observed *L*-proline accumulation. It should be noted that current metabolomic databases have certain limitations, as evidenced by the failure to detect several key intermediates including 6-amino-2-oxohexanoate, P2C, P6C, 1-oxoindolizine, and 1-hydroxyindolizine in our analysis.

### 4.4. SW Biosynthetic Pathway

Based on integrated transcriptomic and metabolomic analyses of *A. oxytropis* OW7.8 and Δ*swnR*, the predicted SW biosynthetic pathway in this study is consistent with our recently proposed model [34]. As more SW biosynthetic genes and their functions continue to be characterized, additional details of the SW biosynthetic pathway in locoweed endophytic fungi will be further elucidated.

## 5. Conclusions

In this study, we successfully cloned and knocked out the *swnR* gene in the locoweed endophytic fungus *Alternaria oxytropis* OW7.8 for the first time, generating the *swnR* knockout mutant Δ*swnR*. Compared with *A. oxytropis* OW7.8, Δ*swnR* exhibited altered colony and mycelial morphology, along with slower growth. After 20 days of cultivation, SW levels in Δ*swnR* were significantly lower than those in *A. oxytropis* OW7.8. In contrast, *swnR*-OE showed a marked increase in SW production relative to Δ*swnR*, confirming that the *swnR* gene functionally promotes SW biosynthesis.

Transcriptomic analysis of Δ*swnR* and *A. oxytropis* OW7.8 identified 2997 DEGs, including 1555 upregulated and 1442 downregulated genes. SW synthesized closely related genes (*P5CR*, *swnN*, *swnK*, *swnH2*, and *swnH1*) that were downregulated, while the *sac* gene was upregulated, consistent with RT-qPCR validation. Metabolomic profiling revealed 1626 differential metabolites between *A. oxytropis* OW7.8 and Δ*swnR*, with five metabolites closely linked to SW biosynthesis: *L*-Lys, *L*-Glutamic acid, Saccharopine, and *L*-Proline were upregulated, and *L*-PA was downregulated.

## Figures and Tables

**Table 1:** Members of the SWN gene cluster and their predicted functions [31].

Gene	Encoding Product	Function Prediction
*swnA*	Aminotransferase	Catalyzing the synthesis of Pyrroline-6-carboxylate (P6C) from *L*-Lysine
*swnR*	Dehydrogenase or reductase	Catalyzing the synthesis of *L*-PA from P6C
*swnK*	Multifunctional protein	Catalyzing the synthesis of 1-Oxoindolizidine (or 1-Hydroxyindolizine) from *L*-PA
*swnN*	Dehydrogenase or reductase	Catalyzing the synthesis of 1-Hydroxyindolizine from 1-Oxoindolizidine
*swnH1*	Fe (II)/α-Ketoglutarate-dependent dioxygenase	Catalyzing the synthesis of SW from 1,2-Dihydroxyindolizine
*swnH2*	Fe (II)/α-Ketoglutarate-dependent dioxygenase	Catalyzing the synthesis of 1,2-Dihydroxyindolizine form 1-Hydroxyindolizine
*swnT*	Transmembrane transporter	Transport of SW

**Table 2:** Identification results of differential metabolites between positive and negative ions.

Screening Mode	Total of Metabolites	Total of DEMs	Upregulated	Downregulated
Positive	1878	984	566	418
Negative	1124	642	439	203

## Data Availability

The data presented in this study are available on reasonable request from the corresponding author.
